# A Fermented Whole Grain Prevents Lipopolysaccharides-Induced Dysfunction in Human Endothelial Progenitor Cells

**DOI:** 10.1155/2017/1026268

**Published:** 2017-03-13

**Authors:** Laura Giusti, Morena Gabriele, Giuseppe Penno, Monia Garofolo, Vincenzo Longo, Stefano Del Prato, Daniela Lucchesi, Laura Pucci

**Affiliations:** ^1^Section of Diabetes and Metabolic Disease, Department of Clinical and Experimental Medicine, University of Pisa and Azienda Ospedaliero-Universitaria Pisana, Via Paradisa 2, 56124 Pisa, Italy; ^2^National Research Council, Institute of Biology and Agricultural Biotechnology (IBBA), Pisa Unit, Research Area of Pisa, Via Moruzzi 1, 56124 Pisa, Italy

## Abstract

Endogenous and exogenous signals derived by the gut microbiota such as lipopolysaccharides (LPS) orchestrate inflammatory responses contributing to development of the endothelial dysfunction associated with atherosclerosis in obesity, metabolic syndrome, and diabetes. Endothelial progenitor cells (EPCs), bone marrow derived stem cells, promote recovery of damaged endothelium playing a pivotal role in cardiovascular repair. Since healthy nutrition improves EPCs functions, we evaluated the effect of a fermented grain, Lisosan G (LG), on early EPCs exposed to LPS. The potential protective effect of LG against LPS-induced alterations was evaluated as cell viability, adhesiveness, ROS production, gene expression, and NF-kB signaling pathway activation. Our results showed that LPS treatment did not affect EPCs viability and adhesiveness but induced endothelial alterations via activation of NF-kB signaling. LG protects EPCs from inflammation as well as from LPS-induced oxidative and endoplasmic reticulum (ER) stress reducing ROS levels, downregulating proinflammatory and proapoptotic factors, and strengthening antioxidant defense. Moreover, LG pretreatment prevented NF-kB translocation from the cytoplasm into the nucleus caused by LPS exposure. In human EPCs, LPS increases ROS and upregulates proinflammatory tone, proapoptotic factors, and antioxidants. LG protects EPCs exposed to LPS reducing ROS, downregulating proinflammatory and proapoptotic factors, and strengthening antioxidant defenses possibly by inhibiting NF-*κ*B nuclear translocation.

## 1. Introduction

Several endogenous signals such as oxidized low-density lipoproteins and exogenous signals such as bacterial lipopolysaccharides (LPS) derived by the gut microbiota orchestrate the early and late low-grade chronic inflammatory responses and trigger and sustain the activation of different innate immune system cells involved in the pathogenesis of atherosclerosis [1]. LPS, often referred to as endotoxin, are fragments from the cell wall of Gram-negative, mostly anaerobic, gut bacteria, a complex ecosystem inhabiting the human gastrointestinal tract. Recent studies have established a clear role for this microbiota including endotoxin in diseases such as obesity, metabolic syndrome, type 2 diabetes, atherosclerosis, and cardiovascular disease (CVD) [2–4]. For instance, the gut microbiota plays an important role in the development and maintenance of the immune system. There is accumulating evidence that many cardiovascular risk factors, both traditional and nontraditional, such as nutrients and altered gut microbiota induce long-term epigenetic reprogramming of cells of the innate immune system. In turn, these changes drive a persistent nonresolving inflammation in the vessel wall and may provoke a condition of continuous innate immune system overactivation, also called innate immune memory, a relatively new aspect in the pathogenesis of atherosclerosis [5]. Notably, low levels of circulating LPS appear to be one of the key culprits in provoking the nonresolving low-grade inflammation [6].

Western type high-fat diets, enriched in polyunsaturated fat and omega-6 fatty acids, are associated with dysbiosis and overgrowth of Gram-negative pathogens, low-grade chronic inflammation in the intestines through activation of NF-kB, and increased intestinal permeability resulting in increased translocation of bacterial LPS and low-grade endotoxemia [7, 8]. Such mainly postprandial low-grade endotoxemia can be defined as “metabolic endotoxemia.” On the contrary, a balanced diet, rich in fibers, keeps balanced microbiota and an intact intestinal barrier function and promotes gut homeostasis. Furthermore, prebiotics and probiotics are examples of dietary manipulation of the gut microbiota [9]. A prebiotic is an ingredient whose fermentation leads to beneficial changes in the gut microbiota and, in turn, to decreased plasma LPS levels through improving the mucosal barrier integrity, decreased metabolic endotoxemia, and reduced low-grade inflammation [9, 10]. Whole grains are concentrated sources of fermentable polysaccharides, insoluble and soluble fiber, resistant starch, and oligosaccharides, which are digested by intestinal microbiota into short-chain fatty acids (SCFAs) that, in turn, act as regulators of food and energy intake and as potent anti-inflammatory agents [11]. A recent meta-analysis reported a consistent and inverse association between whole grains intake and the risk of deaths from all causes, particularly from CVD [12].

Lisosan G (LG) is a fermented powder obtained from organic whole grains* (Triticum aestivum)*. Not only does this product contain the typical components of cereals but also after fermentation it is enriched in bioactive substances such as phenolic components and alpha-lipoic acid [13], a natural antioxidant that can provide protection against reactive oxygen species- (ROS-) induced vascular damage [14]. In healthy rabbits, LG improves lipid profile and antioxidant status [15], while in in vitro studies LG has been shown to exert protective effects in human microvascular endothelial cells exposed to oxidized-LDL controlling both oxidative and inflammatory processes [16]. More recently, protective effects of LG on cell functional properties have been described in human endothelial progenitor cells (EPCs) exposed to oxidative stress. LG enhances EPCs viability and adhesiveness, while reducing their senescence, probably via augmentation of antioxidant defense systems and activation of nuclear factor (erythroid-derived 2)-like 2 (Nrf2) signaling pathway [17].

EPCs, derived from bone marrow and other tissue reservoirs, play a key role in vascular maintenance, reendothelialization, and neovascularization, contributing through correct cells turnover to vascular repair, endothelium integrity, and vessel wall homeostasis [18, 19]. There is growing evidence that dysfunctional EPCs are closely related to endothelial dysfunction, atherosclerosis progression, and the development of various CVD. The underlying mechanisms of EPCs dysfunction, associated with almost all major CVD risk factors, remain largely elusive. So far, it has been well recognized that inflammatory response, disruption of endoplasmic reticulum homeostasis, ROS production, reduced nitric oxide (NO) bioavailability, and their extensive crosstalk are the key factors involved in modulation of EPCs functions under various physiologic and pathologic conditions [20–22].

With this background, the aim of the present study was to evaluate the effects of the prebiotic Lisosan G on functional properties of human early endothelial progenitor cells exposed to oxidative/inflammatory stress induced by lipopolysaccharides; in particular, expressions of several factors involved in cellular inflammatory and oxidative responses, together with the activation of nuclear factor-kB (NF-kB), have been evaluated.

## 2. Materials and Methods

### 2.1. Ethics Statement

Blood samples were obtained from healthy volunteers under written informed consent according to the Declaration of Helsinki. Study protocol was approved by the local Ethics Committee.

### 2.2. Cell Culture

Following overnight fasting, venous blood from healthy volunteers (up to 50 mL) was drawn in EDTA tubes and processed within 2 hours from sampling. Peripheral blood mononuclear cells (PBMCs) were fractionated using Biocoll density-gradient centrifugation (Biochrom AG; density = 1.077 g/mL). PBMCs were seeded on 2 *μ*g/cm^2^ fibronectin coated culture dishes (BD Falcon) or Lab-Tek II chamber slides system (Sigma-Aldrich, Ltd., Poole, Dorset, UK) after erythrocytes lysis. Cells were cultured in endothelial basal medium (EBM-2, Lonza Sales AG, Basel, Switzerland) supplemented with EGM-2-MV-SingleQuots containing human endothelial growth factor, hydrocortisone, insulin-like growth factor, fibroblast growth factor, vascular endothelial growth factor (VEGF), antibiotics, and 5% fetal bovine serum (FBS, Lonza Sales AG). After 3-day culture, nonadherent cells were discarded by washing with PBS and the culture medium was replenished daily. Finally, on day 5, adherent cells, displaying an elongated spindle-shaped morphology, were identified as “early” EPCs.

### 2.3. EPCs Characterization

Early EPCs were characterized from the uptake of 1,19-dioctadecyl-3,3,39,39-tetramethylindocarbocyanine-labeled acetylated low-density Lipoprotein (DiI-Ac-LDL) and lectin binding. Staining was performed by incubating EPCs with 10 mg/mL of DiI-Ac-LDL (Invitrogen, Life Technologies, Ltd., Paisley, UK) for 2 hours at 37°C. Then, cells were fixed in 4% paraformaldehyde for 30 min and counterstained with 1 mg/mL FITC-labelled lectin from* Ulex europaeus* (UEA-1-FITC) (Sigma-Aldrich, Ltd.) for 2 hours at 37°C in the dark. Images of the stained cells were viewed with a fluorescence microscope and double-positive DiI-Ac-LDL/lectin cells were preliminarily identified as early EPCs [23].

To evaluate the immunophenotype of EPCs, adherent cells were detached with trypsin-EDTA and 5 × 10^5^ cell/tube were incubated with anti-human CD34-PE (BD Biosciences), CD133-PE (Miltenyi Biotec), VEGFR-2-Alexa Fluor 647 (BioLegend), CD31-FITC (BD Biosciences), CD45-FITC (BD Biosciences), and CD42-PE (BD Biosciences) antibodies for 30 min in the dark at 4°C. Isotype control antibodies were used to set baseline fluorescence levels. The labeled cells were analyzed on a FACSCalibur Instrument (BD Biosciences), acquiring 2 × 10^4^ events for each analysis. The flow cytometric analysis was repeated six times.

### 2.4. Plant Material

Lisosan G is a powder supplied by Agrisan Company (Larciano (PT), Italy) obtained by fermenting and drying whole wheat flour from* Triticum aestivum* grains. The resulting lysate was extracted with distilled water and sonicated (three cycles: 10 s on/10 s off). Then, the extract was centrifuged for 10 minutes at 2300 ×g at 4°C (Jouan CR3i centrifuge, Newport Pagnell, UK), and the supernatant was collected, filtered (0.2 *μ*m VWR International PBI, Milan, Italy), and kept at 4°C in the dark until use [17].

### 2.5. Study Protocol

To evaluate the effect of LG on EPCs bioactivity, early EPCs were tested into six different experimental conditions:

1. *C*: control experimental condition, that is, cells unexposed to both LG and LPS
2. *LG*: cells incubated for 5 hours or 24 hours with 0.7 mg/mL LG
3. *LPS*: cells treated for 5 hours or 24 hours with 10 *μ*g/mL LPS from* Escherichia coli* (serotype O55:B5, Sigma)
4. *LG + LPS*: after 1-hour pretreatment with 0.7 mg/mL LG, 10 *μ*g/mL LPS were added to the medium and cells were coincubated for further 4 hours or 23 hours with both LG and LPS
5. *1 h LG + LPS*: after 1-hour pretreatment with 0.7 mg/mL LG, the medium was replenished; 10 *μ*g/mL LPS were added to the new medium and cells were incubated for further 4 hours or 23 hours with LPS alone
6. *1 h LG*: cells were incubated for 1 hour with 0.7 mg/mL LG and maintained in the basal medium for further 4 hours or 23 hours.

### 2.6. Analytical Methods

#### 2.6.1. Assessment of Cell Viability

To assess cell viability of cultured EPCs, we used alamarBlue (AB) reagent (Invitrogen, Life Technologies, Ltd.), a nontoxic, cell-permeable compound. Briefly, after 5 days of culture, EPCs (1 × 10^5^ cells/well) were placed on fibronectin-coated 96-well plates and then 10% AB solution was directly added to the medium. As negative control, AB was added to the medium without cells. The plate was incubated for 24 hours at 37°C, 5% CO_2_. The absorbance was quantified at 540 nm (reference wavelength: 620 nm) using a multiplate reader (Multiskan EX, THERMO). The amount of absorbance is proportional to the number of living cells and corresponds to the cell metabolic activity.

#### 2.6.2. Colorimetric Adhesion Assay

Adherent cells were detected by staining with the crystal violet dye. EPCs (1 × 10^5^ cells/well) were placed into fibronectin-coated 96-well plates and exposed to the different experimental conditions. Nonattached cells were gently removed with PBS, whereas adherent EPCs were fixed with 4% paraformaldehyde for 10 min and stained with 0.25% crystal violet for 30 min at room temperature. Excess dye was removed by washing, while the dye absorbed by adherent cells was extracted with 33% acetic acid. Absorbance was read at 540 nm.

#### 2.6.3. ROS Production

ROS production was evaluated by ROS-sensitive fluorescent probe 5-(and-6)-chloromethyl-2′,7′-dichlorodihydrofluorescein, acetyl ester (CM-H2DCFDA) (Invitrogen, Life Technologies, Ltd.). Briefly, EPCs (2 × 10^5^ cell/well) were incubated with CM-H2DCFDA (10 *μ*M/well) for 30 min at room temperature in the dark, and ROS production was detected by measuring the increase in fluorescence recorded at 495 nm excitation and 527 nm emission, using a microplate reader (Varian Cary Eclipse Fluorescence Spectrophotometer).

#### 2.6.4. NF-kB Nuclear Translocation

The activation of NF-kB signaling pathway was evaluated by fluorescence microscopy as NF-kB nuclear translocation after 6, 12, and 24 hours of exposure to 10 *μ*g/mL LPS with or without LG pretreatment. Briefly, EPCs were washed in PBS and fixed with 4% (wt/vol) formaldehyde in PBS for 30 min at room temperature. After washing with PBS, the chamber slides were incubated with 0.2% TRITON X-100 in PBS for 10 min and then blocked with 1% BSA in PBS for 1 hour, followed by overnight incubation with a mouse monoclonal anti-NF-kB p105/p50 IgG-FITC antibody (NB100-78384, Novus Biologicals, Littleton, CO, USA) at 4°C in the dark. Finally, cells were washed with PBS and viewed with a fluorescence microscope. DAPI was used as a nuclei-specific dye.

#### 2.6.5. RT-PCR and Quantitative Real-Time PCR

Total RNA was isolated from EPCs using the NucleoSpin RNA Kit (Macherey-Nagel, Düren, Germany) and reverse-transcribed using the iScript™ cDNA Synthesis Kit (Bio-Rad, CA). Quantitative real-time PCR was performed using the SsoFastTM EvaGreen® Supermix (Bio-Rad, CA) in the StepOnePlus™ Real-Time PCR System (ABI Applied Biosystems, Foster City, CA). Gene primers designed using Beacon Designer Software (PREMIER Biosoft International, USA) were listed in Table 1.

For gene expression analysis of superoxide dismutase 2 (SOD2, Hs00167309_m1), glutathione peroxidase type 1 (Gpx-1, Hs00829989_gH), and heme oxygenase 1 (HO-1 Hs00157965_m1) the predeveloped TaqMan gene expression assays (PE Applied Biosystems) and the TaqMan Universal PCR Master Mix (PE Applied Biosystems) have been employed; eukaryotic 18S rRNA endogenous control (4319413E) was used as housekeeping (PE Applied Biosystems).

All genes were assayed in triplicate and their expression was calculated using a comparative critical threshold (Ct) method. The data are expressed as a fold change of expression levels compared to the control.

### 2.7. Statistical Analysis

Statistical analysis was carried out using SPSS 13.0 software (SPSS Inc., Chicago, IL, USA) for Mac OS X. All data are expressed as means ± SD of at least 3 or more independent experiments. Unpaired Student's* t*-test (two-tailed) was used for single comparisons, while one-way ANOVA with Fisher's Least Significant Difference (LSD) post hoc test was carried out for multiple comparisons.* P* values < 0.05 were considered statistically significant.

## 3. Results

### 3.1. Analysis of EPCs Based on Cell Surface Marker Expression

**Figure 1:**
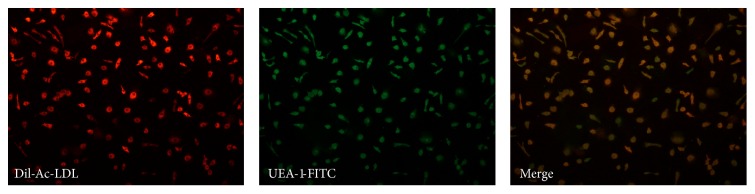
EPCs phenotype characterization by double staining with DiI-Ac-LDL (red) uptake and lectin UEA-1-FITC (green) binding. Merged images showed DiI-Ac-LDL/lectin double-positive EPCs (yellow).

After a 5-day culture under standard conditions, “early” EPCs resulted in an adherent population consisting of cells double-positive for DiI-Ac-LDL and lectin (UEA-1-FITC) as established by fluorescent microscope analysis (Figure 1). EPCs phenotype was confirmed by the expression of the main endothelial cell surface markers such as CD14 (98.90  ±  0.46%), CD31 (41.81 ± 23.17%), CD34 (40.20 ± 32.62%), CD42 (1.18 ± 1.62%), CD45 (96.61 ± 3.71%), CD133 (8.65 ± 6.38%), and VEGFR-2 (43.18 ± 20.35%).

### 3.2. EPCs Viability

**Figure 2:**
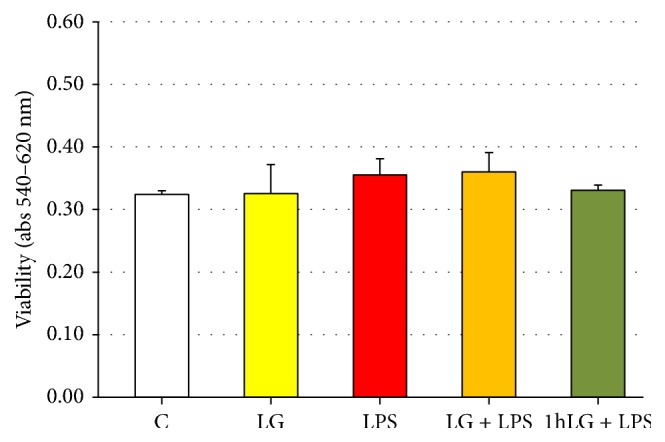
Effects of Lisosan G (LG) on viability of early EPCs in absence or presence of LPS. The absorbance was quantified at 540 nm (reference wavelength: 620 nm). Data are expressed as means ± SD; *n* = 5; *p* = ns.* C*, untreated cells;* LG*, LG-treated cells;* LPS*, LPS-treated cells;* LG + LPS*, 1 h LG-pretreated cells and then coincubated for 4 or 23 hours with LG and LPS;* 1 h LG + LPS*, 1 h LG-pretreated cells and then exposed for 4 or 23 hours with LPS alone.

In order to identify the optimal treatment condition and detect possible direct cytotoxic effects, we firstly performed a toxicity curve using a wide range of LPS concentrations. Thus, to evaluate the effect of LPS on cells viability, early EPCs were incubated for 24 hours with increasing doses (0, 0.01, 0.05, 0.1, 1.0, and 10 *μ*g/mL) of LPS. The highest concentration showing no effect on cells viability (10 *μ*g/mL) was used in all subsequent experiments (cell viability, as determined by AB reagent, was >95%). Conversely, the dose of LG (0.7 mg/mL) was selected based on a previous study [17]. EPCs were treated with 0.7 mg/mL LG and/or 10 *μ*g/mL LPS as previously described in Study Protocol. As shown in Figure 2, at none experimental condition, viability of EPCs treated with LG changed in presence or absence of 10 *μ*g/mL LPS compared to control cells (C).

### 3.3. EPCs Adhesion Capacity

**Figure 3:**
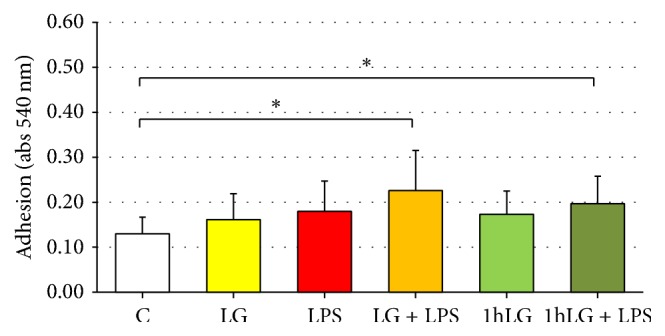
Effects of Lisosan G (LG) on adhesion of EPCs in absence or presence of LPS. Data are expressed as means ± SD; *n* ≥ 5; ^*∗*^*p* = 0.02 versus control (C).* C*, untreated cells;* LG*, LG-treated cells;* LPS*, LPS-treated cells;* LG + LPS*, 1 h LG-pretreated cells and then coincubated for 4 or 23 hours with LG and LPS;* 1 h LG*, 1 h LG-treated cells and then exposed for 4 or 23 hours with basal medium;* 1 h LG + LPS*, 1 h LG-pretreated cells and then exposed for 4 or 23 hours with LPS alone.

No differences were observed as for adhesion ability in EPCs exposed to LG (LG and 1 h LG) or LPS (LPS) compared to untreated cells (C). On the other hand, coincubation with LG and LPS (LG + LPS) and LPS exposure following 1 h LG pretreatment (1 h LG + LPS) increased EPCs adhesion (0.226 ± 0.089 and 0.197 ± 0.061, resp.) compared to control cells (0.130 ± 0.037, *p* = 0.02 for both) (Figure 3).

### 3.4. ROS Production

**Figure 4:**
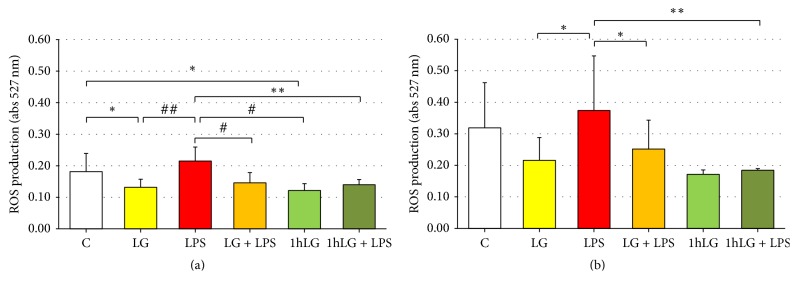
Effects of Lisosan G (LG) on ROS production in absence or presence of LPS. EPCs were incubated for 5 hours (a) and 24 hours (b). Data are expressed as means ± SD; *n* ≥ 5; ^*∗*^*p* < 0.05, ^*∗∗*^*p* < 0.01, ^#^*p* < 0.005, and ^##^*p* < 0.001 or less.* C*, untreated cells;* LG*, LG-treated cells;* LPS*, LPS-treated cells;* LG + LPS*, 1 h LG-pretreated cells and then coincubated for 4 or 23 hours with LG and LPS;* 1 h LG*, 1 h LG-treated cells and then exposed for 4 or 23 hours with basal medium;* 1 h LG + LPS*, 1 h LG-pretreated cells and then exposed for 4 or 23 hours with LPS alone.

Intracellular ROS were measured in early EPCs in the different experimental conditions at both 5 hours (Figure 4(a)) and 24 hours (Figure 4(b)). At 5 hours, compared with untreated cells (0.181 ± 0.058), ROS levels were significantly reduced by LG treatment (LG, 0.131 ± 0.026, and 1 h LG, 0.121 ± 0.022; *p* < 0.05 for both). Conversely, exposure to LPS increased intracellular ROS (0.215 ± 0.044, only marginally versus C; *p* < 0.001 and *p* < 0.005 versus LG and 1 h LG, resp.). This increase in ROS levels was almost fully reverted in cells coincubated or pretreated with LG (LG + LPS, 0.146 ± 0.033, *p* < 0.005; 1 h LG + LPS, 0.140 ± 0.017, *p* < 0.01) (Figure 4(a)). A similar pattern was observed after 24 hours of treatment: the increase in ROS levels induced by LPS (0.374 ± 0.173) was largely reverted in cells coincubated or pretreated with LG (LG + LPS, 0.252 ± 0.092, *p* < 0.05; 1 h LG + LPS, 0.194 ± 0.005, *p* < 0.01) (Figure 4(b)).

### 3.5. Activation of NF-kB

**Figure 5:**
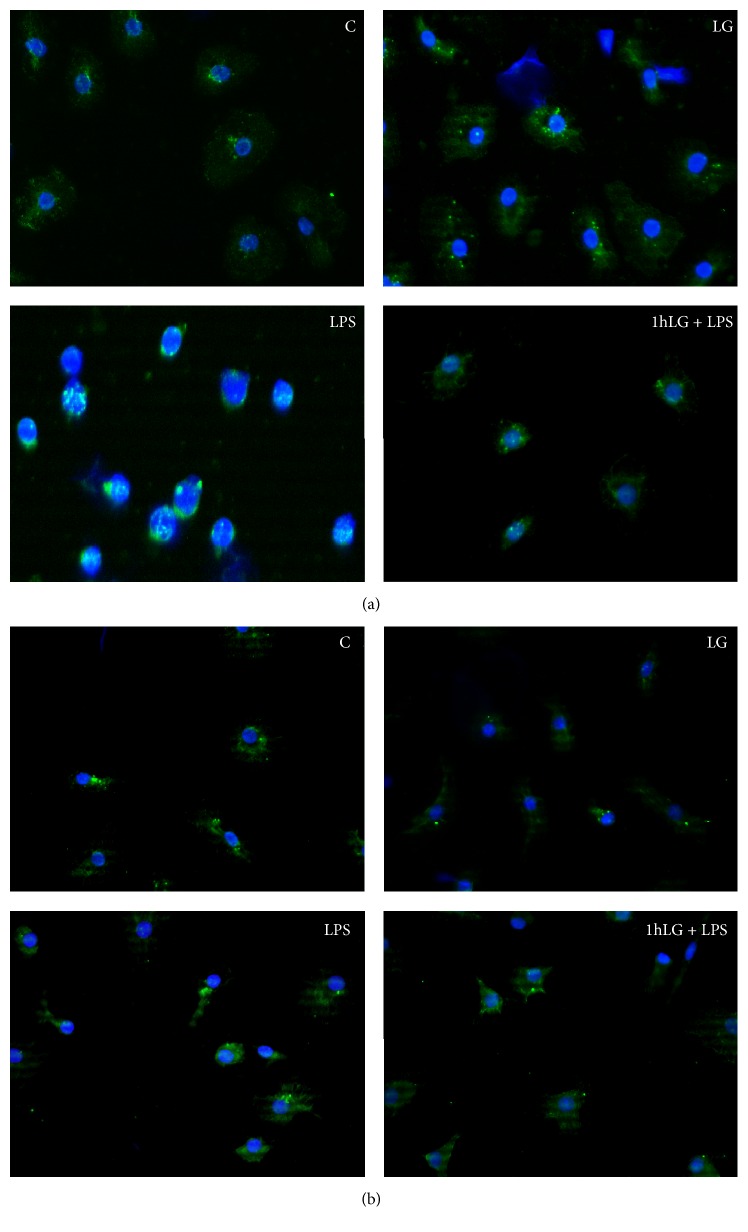
Assessment of NF-*κ*B translocation in the nucleus by fluorescence microscopy. Untreated cells were used as control (C). DAPI was used for nuclei staining (blue), whereas NF-*κ*B was detected as green signal. (a) 12-Hour treatments; (b) 24-hour treatments.* C*, untreated cells;* LG*, LG-treated cells;* LPS*, LPS-treated cells;* 1 h LG + LPS*, 1 h LG-pretreated cells and then exposed for 4 or 23 hours with LPS alone.

NF-kB is an oxidant sensitive transcription factor responsible for regulating gene expression offactors involved in inflammatory response, apoptosis, and immune and stress responses. Herein, we explored whether LG prevents NF-*κ*B translocation from the cytosol into the nucleus caused by exposure to LPS for 6, 12, and 24 hours. As shown in Figure 5, in control cells and in cells exposed to LG, NF-*κ*B is mainly localized in the cytosol. Conversely, LPS treatment activates NF-kB signaling pathway, through NF-kB translocation, at all time points, mainly after 12-hour exposure (Figure 5(a)) and to a lesser extent after 24-hour exposure (Figure 5(b)), whereas 1-hour LG pretreatment fully prevents NF-*κ*B nuclear translocation both at 12 and 24 hours.

### 3.6. Gene Expression of Proinflammatory and Proapoptotic Factors

**Figure 6:**
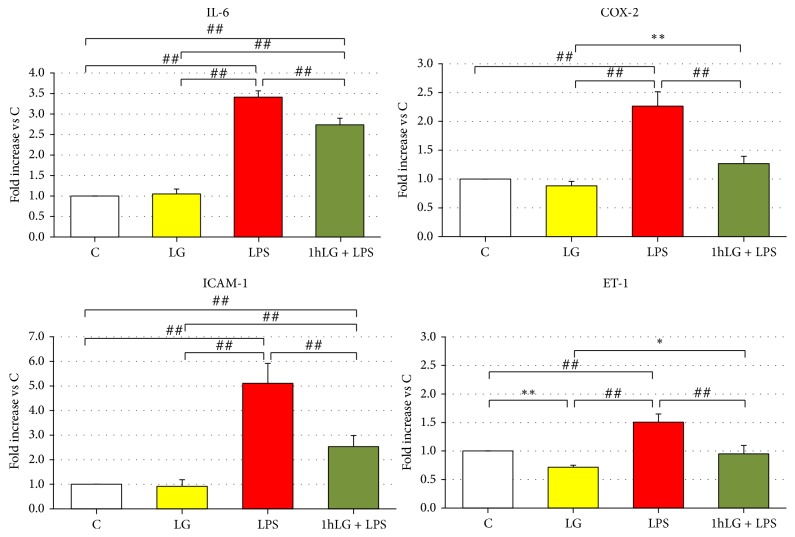
Effects of 0.7 mg/mL LG pretreatment on EPCs exposed for 23 hours to 10 ug/mL LPS: quantitative real-time RT-PCR analysis of IL-6, COX-2, ICAM-1, and ET-1 gene expression. The bars represent mean ± SD fold increase in transcript expression relatively to untreated cells (C); *n* ≥ 3; ^*∗*^*p* < 0.05, ^*∗∗*^*p* < 0.01, and ^##^*p* ≤ 0.001 or less.* C*, untreated cells;* LG*, LG-treated cells;* LPS*, LPS-treated cells;* 1 h LG + LPS*, 1 h LG-pretreated cells and then exposed for 4 or 23 hours with LPS alone.

**Figure 7:**
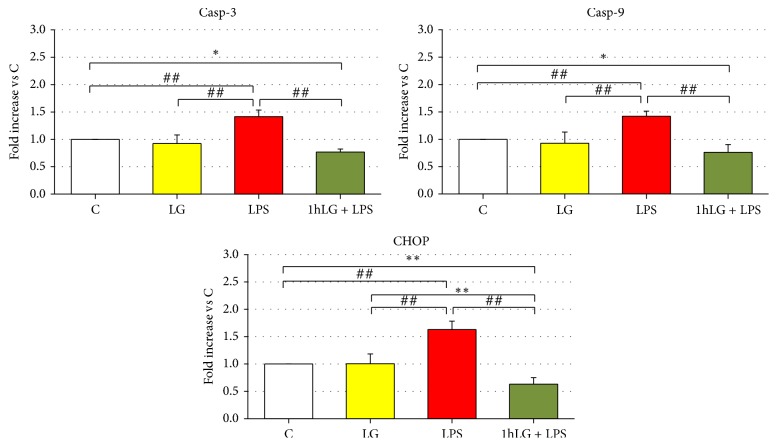
Effects of 0.7 mg/mL LG pretreatment on EPCs exposed for 23 hours to 10 ug/mL LPS: quantitative real-time RT-PCR analysis of Casp-3, Casp-9, and CHOP gene expression. The bars represent mean ± SD fold increase in transcript expression relatively to untreated cells (C); *n* ≥ 3; ^*∗*^*p* < 0.05, ^*∗∗*^*p* < 0.01, and ^##^*p* ≤ 0.001 or less.* C*, untreated cells;* LG*, LG-treated cells;* LPS*, LPS-treated cells;* 1 h LG + LPS*, 1 h LG-pretreated cells and then exposed for 4 or 23 hours with LPS alone.

Specifically, we investigated at 24 hours gene expression of proinflammatory and proapoptotic factors in early EPCs exposed to LG alone (LG) and LPS alone (LPS) and pretreated for 1 hour with LG and then exposed to LPS (1 h LG + LPS), as compared to control cells (C). LG exposure (LG) did not affect gene expression of IL-6, ICAM-1, and COX-2 as well Casp-3, Casp-9, and CHOP. Only ET-1 mRNA was slightly reduced (*p* = 0.01) as compared to C. Conversely, LPS (LPS) caused a significant increase in gene expression of all proinflammatory and proapoptotic factors as compared to both C and LG (Figures 6 and 7). 1 h LG exposure significantly reduced without normalizing IL-6 (*p* = 0.0002) and ICAM-1 (*p* < 0.0001), almost normalized mRNA for COX-2 and ET-1, and even significantly reduced Casp-3, Casp-9, and CHOP gene expression (*p* < 0.05 or less) compared to C.

### 3.7. Gene Expression of Antioxidant Enzymes

**Figure 8:**
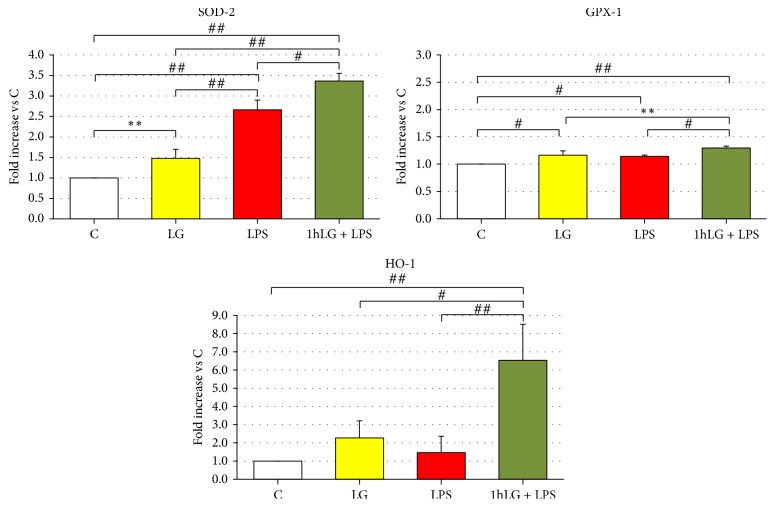
Effects of 0.7 mg/mL LG pretreatment on EPCs exposed for 23 hours to 10 ug/mL LPS: quantitative real-time RT-PCR analysis of SOD-2, GPX-1, and HO-1 gene expression. The bars represent mean ± SD fold increase in transcript expression relatively to untreated cells (C); *n* ≥ 3; ^*∗∗*^*p* < 0.01, ^#^*p* < 0.005, and ^##^*p* < 0.001 or less.* C*, untreated cells;* LG*, LG-treated cells;* LPS*, LPS-treated cells;* 1 h LG + LPS*, 1 h LG-pretreated cells and then exposed for 4 or 23 hours with LPS alone.

Behaviour of antioxidant enzymes gene expression resulted to be more heterogeneous (Figure 8). Compared to C, SOD-2 mRNA was slightly increased in LG (*p* = 0.01) and 2.5 and more than 3 times higher in LPS and 1 h LG + LPS, respectively (*p* < 0.0001 for both). GPX-1 showed a similar pattern but with only a slight though significant increase between different experimental conditions. Finally, HO-1 was significantly higher in 1 h LG + LPS (*p* = 0.002 or less) than in all other experimental conditions.

## 4. Discussion

The switch from a healthy to a dysfunctional endothelium is widely recognized as the earliest step in the development of atherosclerosis and its prevention represents an attractive target to improve cardiovascular outcomes. An appealing way to reach this objective is to sustain the continuous replacement of lost or dysfunctional endothelial cells by preserving the reendothelialization process operated by the endothelial progenitor cells at sites of endothelial injury [24]. “Early” endothelial progenitor cells, also termed CAC (circulating proangiogenic cells) display low proliferative potential and low propensity to integrate as long-lived endothelial cells but promote vascular repair mainly in a paracrine manner via production of proangiogenic factors and cytokines [25]. Likely, “early” EPCs represent a first wave in endothelial regeneration process [24].

ROS are intrinsic regulators involved in maintaining the abilities of self-renewal of stem cells and their differentiation into “lineage-committed” progenitor cells [26]. Furthermore, ROS are involved differently in the modulation of stem and progenitor cell availability and function under various physiologic and pathologic conditions [20]. The levels of ROS are the result of the harmony between ROS generation, largely mediated by NADPH oxidase (NOX) [20], and antioxidant defense systems [26]. In general, low levels of NOX-derived ROS function as a signal that mediates EPCs proliferation, mobilization, migration, and differentiation [20]. Under pathological conditions, increased oxidative stress not only reduces circulating EPCs levels but also impairs EPCs functions [20].

Recent studies have indicated that ROS mediate the LPS-induced proinflammatory response and its unrestricted cytokines production, with mitochondrion as the major intracellular source of LPS-stimulated ROS production [27, 28]. Furthermore, the inhibition of ROS generation modulates the production of proinflammatory cytokines by preventing LPS-induced mitogen-activated protein kinase (MAPK) and NF-kB pathways activation.

For the first time, the present study demonstrated in human “early” EPCs that exposure to LPS (LPS experimental condition) increases intracellular ROS production and upregulates expression of both proinflammatory cytokines and proapoptotic factors, while increasing expression of antioxidant enzymes. On the other hand, treatment of EPCs with the prebiotic Lisosan G, a fermented powder from organic whole wheat grains [13, 16, 17] (LG and 1 h LG experimental conditions), reduces intracellular ROS, without affecting expression of both proinflammatory and proapoptotic factors; expression of antioxidant enzymes was also significantly increased. Interestingly, we have observed that ROS levels were reduced to the same extent in EPCs continuously exposed to LG (LG) and in EPCs pretreated with LG (1 h LG) and that those changes were consistent when EPCs were examined at 5-hour and 24-hour exposures (Figures 4(a) and 4(b)). Finally, LG protects EPCs exposed to LPS reducing ROS, lowering or normalizing cytokines, reducing proapoptotic factor even below levels observed in basal experimental conditions (C), and strengthening the whole spectrum of antioxidant defenses herein explored. Once again, ROS levels were reduced to the same extent in EPCs continuously coexposed to LG and LPS (LG + LPS) and in EPCs pretreated with LG and then exposed to LPS (1 h LG + LPS); changes were consistent at 5-hour and 24-hour exposures.

In vitro stimulation of cultured cells by LPS is a widely used tool to study the ability of cells to become activated. Endotoxin elicits a variety of proinflammatory responses pertinent to mechanisms of atherosclerosis. In the three major types of resident vascular cells (endothelial cells, smooth muscle cells, and fibroblasts), treatment with LPS results in upregulation of antioxidant enzyme activity, consistent with increases in oxidative stress. Both these findings, increased ROS production and upregulation of antioxidant defenses, have been confirmed by our study in human EPCs exposed to LPS. NADPH oxidase activation and MAPK signaling pathways play critical roles in the expression of LPS-enhanced Toll-like receptor 4 (TLR-4). TLR-4, a proinflammatory sensor of pathogens, in turn, contributes to fueling low-grade vascular inflammation [29]. This response was found throughout the vessel wall rather than being localized to a single cell type. Indeed, endotoxin contributes directly but mainly indirectly throughout elevation of ROS to increased release of a large number of inflammatory cytokines and other effectors, including interferon-*γ*, IL-1 and IL-6, and tumor necrosis factor-*α* (TNF-*α*) along with granulocyte-macrophage colony-stimulating factor and platelet-activating factor [30]. Furthermore, endotoxin upregulates expression of a number of chemokines that act as chemoattractans and leukocytes activators (monocytes chemoattractant protein-1 and IL-8) and adhesion molecules (selectins, ICAM-1, VCAM-1, and platelet and endothelial cell adhesion molecule-1 (PECAM-1)). Interestingly, PECAM-1 plays a critical role in the maintenance of human vascular endothelial barrier function [31]. In our study, human EPCs exposed to LPS showed significantly increased expression of both IL-6 and ICAM-1 together with upregulation of both COX-2 and ET-1. When the increase of ROS was inhibited by LG, the production of proinflammatory cytokines in response to LPS was blunted.

COX-2 is an inducible endothelial prostaglandin synthase whose expression increases in response to inflammatory insult; in endothelial cells, the generation of an array of COX-2-derived vasoconstrictive prostanoids is associated with the occurrence of endothelium-dependent contraction, elevated arteriolar tone, and impaired vasodilatation [32]. COX-2 plays an important role in atherosclerosis. Its upregulation is related to the proinflammatory and proatherosclerotic effects of smoking on endothelial cells. Human atherosclerosis is associated with the expression of COX-2 in several vascular beds, mainly in unstable compared with stable lesions; COX-2 is predominantly localized in macrophages within these lesions [33], especially in the shoulder region of plaques, while its expression in native endothelium is still debated [32, 33]. Indeed, COX-2 seems not expressed by the native cells of umbilical veins (HUVECs) and only appears during cell culture [33]. To our knowledge, our study is the first one to show expression of COX-2 in human “early” EPCs under basal conditions; however, the possibility that also for EPCs COX-2 expression might be the result of culture cannot be ruled out. In other studies, expression of COX-2 in EPCs was almost undetectable at baseline but increased after treatment with TNF-*α*, and the effect on COX-2 expression was greater than that of human endothelial cells [34, 35].

ET-1 is a marker of endothelial injury and dysfunction which mainly has a role as a potent endogenous vasoconstrictor secreted by the endothelial cells. ET-1 induces proinflammatory mechanisms in the vascular wall, increasing superoxide anion production, cytokine secretion including IL-1, IL-6, and TNF-*α*, and enhancing the expression of adhesion molecules. It is involved in the activation of transcription factors such as NF-*κ*B [36]. In our study, ET-1 expression in human “early” EPCs increased 1.5-fold upon exposure to LPS being normalized by pretreatment with LG (1 h LG + LPS experimental condition). The putative role of these changes remains unclear. Indeed, in a previous study involving “early” EPCs from healthy men, ET-1 does not affect EPCs colony formation, migratory capacity, or release of vascular endothelial growth factor and granulocyte-colony stimulating factor but does increase EPCs susceptibility to apoptosis, suggesting a role in the proatherogenic effects of ET-1.

Herein, the potential protective effect of LG against LPS-induced changes in “early” EPCs has been evaluated not only in terms of ROS production and expression of proinflammatory factors but also for the expression of proteases playing essential roles in programmed cell death (caspases) and transcription factors involved in endoplasmic reticulum (ER) stress-induced apoptosis (CHOP) [37, 38]. Recently, CHOP has been proposed as a key molecule not only in apoptosis but also in metabolic and inflammatory processes. Indeed, the treatment of macrophages with LPS induces the expression of CHOP that, in turn, mediates the production of interleukins through caspases activation [37]. In our study, exposure to LPS increases expression of both Casp-3 and Casp-9 as well as that of CHOP, while 1-hour pretreatment with LG blunted all proapoptotic factors to levels even lower than those observed in basal experimental conditions. Changes in both proinflammatory molecules and proapoptotic factors are contrasted by consensual modifications in the whole array of antioxidant defenses herein explored. Indeed, expressions of SOD-2, GPX-1, and HO-1 are significantly enhanced in EPCs exposed to LPS with a further increase in cells exposed to LPS upon pretreatment with LG.

ER stress, oxidative stress (ROS), and inflammatory responses compose the major defense networks that allow cells to adapt to and survive stress conditions imposed by a large array of physiological and pathological stimuli. The signaling pathway activated by disruption of ER homeostasis, known as unfolded protein response (UPR), is linked with ROS production, inflammation, and apoptosis [39]. Accumulating evidence suggests an extensive crosstalk between ER stress, oxidative response, inflammatory cytokines, and apoptosis in cells. For instance, it has been described that CHOP, an ER stress-responsive transcription factor with apoptotic function, is involved in the inflammatory processes and plays crucial roles in the development and progression of atherosclerotic diseases [40, 41]. Furthermore, nuclear factors Nrf2 and Nrf1, two oxidative stress-induced transcription factors responsible for regulating the expression of many antioxidant genes, can also be activated by ER stress and UPR machinery to compensate stress condition, thus extending the crosstalk to include the activation of the antioxidant defense system [42]. Among the Nrf2 target genes are phase II antioxidant proteins including HO-1. LPS has been shown to induce the antioxidant enzyme HO-1 through activation of Nrf2, the master regulator of the antioxidant response [43]. Interestingly, in our study, HO-1 shows an about 6-fold increase upon exposure to both LG and LPS (1 h LG + LPS experimental condition) but not when EPCs were exposed to LPS alone. Recent studies demonstrate that Nrf2 activity is suppressed by NF-*κ*B at transcriptional level [44].

NF-*κ*B is a pleiotropic transcription factor responsible for regulating gene expression of molecules involved in inflammation, apoptosis, and immune and stress response [21]. LPS, through ROS overproduction, are among the most powerful NF-*κ*B activator [45]. While in an inactive state NF-*κ*B is located in the cytosol, the activated form is translocated to the nucleus where the transcription of genes encoding proinflammatory cytokines and chemokines takes place [21]. This is exactly what it has been demonstrated in our study: LPS treatment activates NF-*κ*B signaling pathway, that is, its translocation to the nucleus, whereas LG pretreatment fully prevents NF-*κ*B translocation. We are tempted to speculate that, within a complex interplay between NF-*κ*B and Nrf2, the dampening of the antagonistic effects of NF-*κ*B on the Nrf2 pathway exerted by LG pretreatment allows the upregulation of the antioxidant defense system and offers an explanation of the particular course of HO-1 expression.

In a previous study, we reported that LG 0.7 mg/mL improved EPCs viability and adhesion capacity compared to control cells, with LG protecting EPCs against H_2_O_2_-induced toxicity by restoring both viability and adhesion [17]. In the present study, compared to controls, EPCs viability did not change in any experimental condition, while cells adhesiveness, unaffected by LG or LPS, was enhanced by coincubation with LG and LPS (LG + LPS) and in cells exposed to LPS upon pretreatment with LG (1 h LG + LPS). In EPCs from mice, LPS induced cell proliferation indicating that LPS activates EPCs rather than inhibiting them [46]. Another study also showed that LPS treatment unexpectedly failed to induce human EPCs apoptosis, instead promoting cell proliferation [47]. These data look consistent with our observation of preserved viability in human “early” EPCs exposed to LPS. In EPCs from mice, the adhesion capacity was impaired by LPS and showed dose-dependence and time-dependence [46]. In our study, adhesiveness was assessed only at one time point; thus the possibility that we have lost the time-dependent course of changes in EPCs adhesiveness cannot be ruled out. Anyway, in our study, adhesion capacity of “early” EPCs was strengthened by treatment with LG. Moving from “early” EPCs to endothelial colony-forming cells (ECFCs), also called “late” EPCs, it is worthy to mention that incubation with LPS inhibited the viable, migratory, adhesive, and in vitro vasculogenesis capacity of ECFCs isolated from human umbilical cord blood [48].

This work shows for the first time that Lisosan G, a bioavailable antioxidant-rich food supplement, improves EPCs functional properties under the inflammatory insult resulting from LPS exposure, thus counteracting the harmful effect induced by inflammation on the endothelium.

Indeed, our study suggests that exposure of human “early” EPCs to LPS starts up an extensive crosstalk between ER stress, ROS overproduction, and upregulation of proinflammatory and proapoptotic responses integrated with the activation of the antioxidant defense system. Also, we demonstrate that, in EPCs exposed to LPS, Lisosan G restrains or normalizes the proinflammatory response, downregulates caspases and other components of the ER stress-mediated apoptosis pathways, and contrasts efficiently ROS production, further strengthening the activation of the antioxidant defenses. Furthermore, we observe that Lisosan G reduces ROS production and activates the antioxidant enzyme system also at basal conditions, not only upon exposure to LPS. Lastly, Lisosan G prevents the LPS-induced activation of the NF-*κ*B signaling. Thus, Lisosan G joins other natural compounds with antioxidant properties being able to improve overall bioactivity of EPCs exposed to inflammatory insults by resetting the crosstalk between several components of the adaptative intracellular network [49].

## Figures and Tables

**Table 1:** 

Gene	Forward 5′-3′	Reverse 5′-3′
Interleukin-6 *(IL-6)*	AAAGCAGCAAAGAGGCAC	TTCACCAGGCAAGTCTCC

Cyclooxygenase-2 *(COX-2)*	CCGAGGTGTATGTATGAGTGT	CTGTGTTTGGAGTGGGTTTC

Intracellular adhesion molecule-1 *(ICAM-1)*	ACCGTGAATGTGCTCTCC	TCTTGATCTTCCGCTGGC

Endothelin-1 *(ET-1)*	GCAGAAACACACAGTCACAT	TCAGACACAAACACTCCCTTA

CCAAT-enhancer-binding protein homologous protein *(CHOP)*	GAGAGTGTTCAAGAAGGAAGTGTA	CCCGAAGGAGAAAGGCAAT

Caspase-3 *(Casp-3)*	GCTGCCTGTAACTTGAGA	TGCGTATGGAGAAATGGG

Caspase-9 *(Casp-9)*	GAGACCCTGGACGACATC	TTCACCGAAACAGCATTAGC

*β*-*Actin*	GAGATGCGTTGTTACAGGAAG	TGGACTTGGGAGAGGACT
